# Anaesthesia.—New Researches, Statistical and Chemical, Confirming the Duty to Use Ether Instead of Chloroform

**Published:** 1868-01

**Authors:** J. E. Petrequin, B. E. Cotting

**Affiliations:** Ex-Surgeon-in-chief of the Hotel Dieu at Lyons, &c., &c.; Roxbury.


### ARTICLE IX.

### *Anaesthesia.—New Researches, Statistical and Chemical, Confirming the Duty to Use Ether instead of Chloroform.*

By J. E. Petrequin, Ex-Surgeon-in-chief of the Hotel Dieu at Lyons, &c., &c.

(Translated and abridged from the French in *L'Union Medicale*, for the Boston Medical and Surgical Journal, by B. E. Cotting, M.D. of Roxbury.)

The choice between ether and chloroform for anæsthetical purposes must rest upon experience. In the history of the two, ether has made notable progress—both in methods of administration and in purification. Complete anæsthesia can be produced by it with certainty and safety. It is not so with chloroform! No method of administration has removed its dangers, and no purification of the agent has secured safety. In fact, it has not made progress in either respect, and still continues year by year to furnish its numbers of victims.

Inasmuch, therefore, as ether is equally effective in all cases, and has not the dangers of chloroform, it is, in strict logic, an imperative duty to use ether instead of chloroform as an anæsthetic.

This conclusion, apparently incontrovertible, has been attacked by a distinguished surgeon, who declares that "pure chloroform, properly administered, never kills." Now this is a mere assertion, which cannot stand in the face of known facts. And, first, when among those who have met with mishaps we find names prominent in science, for example, MM. Robert, Manec, Marjolin, Richet, Fano, Jarjavay, &c., in Paris; MM. Barrier and Bonnet, to say nothing of other colleagues in Lyons; M. Languebeck, in Germany; MM. Lawrence, Gore, Lane, Bryant, &c., in England; who will dare to say that these men did not know how to administer chloroform? Are not these names in themselves a demonstration that there is no foundation for the pretended infallibility of the rules for its administration? Then, again, secondly, it will not do to assert that every accident presupposes *impurity*, without telling us in what the impurity consists. For, on the contrary, where accidents have happened, time and again, as in instances we cite,* the chloroform has been analyzed by most eminent chemists and found perfectly pure.

---

*In order to condense this paper, the citations in the original are omitted in the translation, and the conclusions only given. In one disastrous case, however, chemical analysis demonstrated the purity of the chloroform, which had been taken from a flask used successfully but a few moments before by a young girl.

Thus then, on the one hand, it has not been shown that the presence of extraneous matters in the chloroform has been the cause of death; while, on the other, is has been proved that its perfect purification has not in any degree diminished such catastrophes.

Thus much established, nothing will better complete the parallel between ether and chloroform, or better determine the choice which ought to be made between them, than a comparative examination of the impurities they are each likely to contain, and their liability to produce disaster.

To begin with ether: it is well known that ether is the product of the reaction of sulphuric acid upon alcohol. Its principal impurities arise in the process itself. Thus ether may contain water; hydrated alcohol, heavy oil of wine, empyreumatic oils, sulphurous acid, and at times a trace of sulphuric acid, &c. The oil of wine may decompose, and give to ether an acid reaction, which has been attributed to sulphurous acid. Air in the flasks containing ether may also, in time, cause further decompositions, as into water and acetic acid. The oil of wine, and, above all, empyreumatic oils and sulphurous acid, take from ether its penetrating and agreeable odor, and its punget and aromatic taste. Shaken with water, ether makes a turbid mixture if it contains oil of wine or pyrogenous oils. The presence of acetic, sulphurous, and traces of sulphuric acids may be detected by their reddening litmus paper. Sulphurous acid is especially recognizable by its action on the salts of barytes.

To purify ether, let it be washed in water which takes up, or, better, in a weak solution of caustic potash, which saturates all the acids; then distil it over lime from a water-bath, it being so volatile that it passes over first, at 96.5° Fahr., leaving behind the heavy oil of wine, water, hydrated alcohol, and the acids converted into salts by the potash. If below proper standard (63° to 65°, aréomètre de Baumé,) it may be submitted to a second distillation.

Thus, as any one may see, there is nothing very deleterious here. These impurities may render etherization laborious, disagreeable, complicated with nausea and nervous excitation, but are not in themselves essentially dangerous. Besides, all these impurities may be removed, by washing, and subsequent distillations carried to the required rectification.

To continue the examination comparatively; it is well known that chloroform is the result of the reaction of chlorinated lime on alcohol. Its principal impurities arise in the process itself, and from spontaneous decomposition. It may contain alcohol, chlorous water, hypochlorous and chlorohydric acids, ether, hydrocarbonated oils, aldehyd, fixed substances, &c.

To obtain chloroform pure, MM. Pelouse and Frémy (*Traite de Chimie*, t. v., 1865) state that it is sufficient to wash it in water, and distil with a water-bath. MM. Barreswill and A. Girard (*Dict. de Chimie Industr.*, t. ii., 1862) first wash the chloroform with water containing carbonate of soda, to remove the chlorine, aldehyd, alcohol, and to saturate acids. On being allowed to rest, the chloroform separates and sinks to the bottom of the liquid. They then decant, wash anew until it reaches 48° (aréomètre,) and then carbonize the oils and organic impurities with sulphuric acid, which is also removed by a final washing; after which they distil the chloroform from the water-bath over a solution of carbonate of soda, to saturate any acids that may have previously escaped removal.

The importance of the question at issue determined M. Pétrequin to institute with M. Emile Chevalier, a pharmaceutical chemist at Lyons, a series of experiments, of which he gives the detailed results in the *Union*.* He concludes:—

Our experiments show that chloroform of commerce does not contain alcohol, ether, chlorine, hydrochloric or

* Omitted in the translation for sake of brevity; so, also, statistics of casualities, more than two hundred, afterwards enumerated by the author.

hypochlorous acid. It has a little formic and acetic acids, and perhaps a slight trace of aldehyd. As to the compounds of methule, the great dangers of which have been bruited so loudly, we repeat here that as M. Letheby, who called attention to them, himself avows that there are no reagents in chemistry capable of detecting such compounds, it is prudent to wait before final decision. Besides, a double washing is sufficient to remove them, if present.

In conclusion, it is evident that the danger lies in chloroform itself. If it kills, it is not because it is impure; *it is because it is in its nature a poison*—a fact shown unquestionably in experiments upon animals, as well as in human pathology.

In the actual state of science, then, the only way to avoid the censure of society, to secure the protection of justice, and, above all, to remain at peace with one's own conscience, is to discard forever a dangerous agent, which, every time that it is used, puts at hazard the fearful question of life or death.*

---

### MONTHLY SUMMARY.

---

*Food for Babies.*—From an interesting article on "Food for Babies," published in the London *Medical Times and Gazette*, we make the following extracts.—*Boston Med. and Surg. Journal.*

Of milk, we have that of the ass, goat, and cow. Asses' milk is by general consent the best substitute for the woman's for most delicate children; and, dear as it is, it is well worth the money. The goat's is a rich milk, and with a strong curd, and

---

* Appropriate to our subject are the memorable words of M. Amedee Latour:—"In face of disasters constantly accumulating, in presence of desolating fatalities increasing in number day by day, is it allowable to remain inflexible in positions, heretofore reasonable perhaps, *but which events too sad and numerous now requires us to modify?* What is a position, be it ever so legitimate, compared with the life of the most insignificant of men? And is it not the absolute and supreme respect for human life which gives grandeur and dignity to our art?"

